# Fracture of Three Bicuspid Teeth Longitudinally by Contrecoup

**Published:** 1872-10

**Authors:** Paul F. Eve

**Affiliations:** Nashville, Tennessee.


### ARTICLE VIII.

### *Fracture of Three Bicuspid Teeth Longitudinally by Contrecoup.*

BY PAUL F. EVE, M. D., NASHVILLE, TENNESSEE.

In the twenty-seventh volume of this journal, 1870, may be found the account of two unusual fractures—one of the anterior superior spinous process of the ilium, and the other of the sternum. Attention is now called to one still more curious and infrequent.

On the 25th of July, my friend and neighbor, Dr. G. W. Currey, brought to my office Mr. Cotter, a brakeman on the Northwestern railroad, who, in attempting to check the progress of an engine at a station near the city, by putting the end of a fence-rail under the wheels, was violently struck under the chin by the other end being thrown up, by which he was rendered insensible for half an hour. The patient was in good health, and aged 25 years. After a few days, he came to Nashville, and was seen by Dr. C., who said to me, "Here is a singular case of fracture, and I want you to report it, for it will require witnesses to make even the profession believe it." The two left bicuspids and the first one of the right side were seen to be fissured, perpendicularly, and vertically, apparently to the extremity of their fangs. The inner halves could be pressed inwards by the probe or nail of the finger, and were moreover quite loose. The man had suffered greatly, but now permitted these loose portions to be removed, which confirmed the supposition of their division down to the extremity of the roots.

The *rationale* of the accident we think was this—that as the superior dental arch overlaps somewhat the inferior, the outer cusps of the lower jaw were, by the blow, driven between those of the corresponding and antagonist in the superior, and like a wedge, had split them in two. The integuments under the chin were contused, but there was no fracture of the inferior or superior maxillary.

That the breaking of a bicuspid tooth longitudinally is rare occurence, may be inferred from the fact that in the recent work on Oral Diseases and Surgery, by Prof. Garretson, M. D., &c., edition of 1869, no other allusion is made to the subject than to those which take place in attempts at their extraction; and Mr. John Tomes, F. R. S., Dentist to the London and Middlesex Hospitals, probably the highest English dental authority, states that the molars may be injured by the jaws being violently driven together, so that their cusps, or spear-like processes, may be broken off. He mentions a case in which he had seen the fissure extend from the crown through the greater portion of the root. The only one somewhat similar to that reported here, is met with in vol. x., *Dictionnaire De Medicine*, in thirty volumes, in which it is said that M. Duval once saw a man of sixty years who had two bicuspids of the superior maxillary fractured longitudinally, without much effort or pain. The author of the article, M. Oudet, states also that he had met with two or three like cases.

In the instance now recorded, both superior small molars on one side, and the first of the other side, were simultaneously split from their crowns to the ends of their fangs, by a vlolent blow suddenly received under the chin. The pulp cavities having been fully exposed, the teeth will necessarily be lost —*Nashville Jour of Med. & Surg.*

---

